# 液体活检在肺癌早期诊断中的研究进展

**DOI:** 10.3779/j.issn.1009-3419.2018.08.08

**Published:** 2018-08-20

**Authors:** 治鹏 宋, 洋 刘

**Affiliations:** 101149 北京，首都医科大学附属北京胸科医院流行病研究室 Department of Epidemiology, Beijing Chest Hospital, Capital Medical University, Beijing 101149, China

**Keywords:** 肺肿瘤, 液体活检, 诊断, 循环肿瘤细胞, 循环肿瘤DNA, 微小核糖核酸, 外泌体, 肿瘤血小板, Lung neoplasm, Liquidbiopsy, Diagnosis, Circulating tumor cells, Circulating tumor DNA, MicroRNA, Exosomes, Tumor educated platelets

## Abstract

肺癌的早期诊断有利于提高患者的生存率。应用影像学方法对肺癌高风险人群进行筛查，可以起到早发现、早诊断的作用。越来越多的研究显示，液体活检（liquid biopsy）可以对该方法进行替代和补充。检测肺癌患者外周血中的循环肿瘤细胞（circulating tumor cells, CTCs）、循环肿瘤DNA（circulating tumor DNA, ctDNA）、微小核糖核酸（microRNA, miRNA）、外泌体（exosomes）、肿瘤血小板（tumor educated platelets, TEPs）可以用于肺癌的早期诊断，并且可能为影像学检查阴性的高风险人群提供相应的诊疗建议。全文就以上标志物的检测手段、在肺癌早期诊断中的价值以及存在优势与局限性进行综述，以期促进液体活检在肺癌早期诊断、与其他筛查手段相结合方面的应用。

## Abstract (en)

The early diagnosis of lung cancer can improve the survival rate of patients. Using imaging method to screen high-risk population plays an important role in early detection and early diagnosis. More and more research shows that liquid biopsy can replace and supplement the method. Detection of circulating tumor cells (CTCs), circulating tumor DNA (ctDNA), microRNA (miRNA), exosomes, and tumor educated platelets (TEPs) in patients' peripheral blood can be used for the early diagnosis of lung cancer, and may provide appropriate medical advice for high-risk population with negative imaging finding. The full text reviews the detection methods of these markers, their value in the early diagnosis, as well as their advantages and limitations, in order to promote the application of liquid biopsy in the early diagnosis and other fields.

肺癌是导致世界范围内癌症死亡人数最多的疾病。因此，对肺癌高危人群（年龄在55岁以上、重度吸烟者、慢性阻塞性肺病患者、肺癌家族史等）开展相应的临床试验项目，即对肺癌进行早期诊断就显得格外重要。目前，试图建立这种早期诊断或筛查的方法有两种：其一是应用影像学手段——低剂量螺旋计算机断层扫描（low-dose spiral CT, LDCT），其二是应用液体活检技术检测循环或非循环生物标志物。这两种方法可能是相辅相成的。在简要介绍影像学手段的局限后，本文将重点阐述液体（血液）活检技术在肺癌早期诊断中的研究进展。

## 1 应用影像学对肺癌进行早期诊断和筛查

相关研究^[1-7]^证明LDCT可用于肺癌的早期诊断。随访肺癌高危人群后发现，该方法可以筛选出一定数量的无症状肺实性结节患者，随后对该人群中疑似肺癌的进行病理诊断并且手术，最终大大降低了筛查人群的死亡率^[3]^。但也有研究^[1, 4, 7]^显示，少数肺癌高危个体因自身差异导致受益并不显著。揭示了影像学方法作为筛查手段的局限性^[8]^。

欧洲国家并未建议应用影像学手段来进行肺癌筛查^[8]^。可能的原因是，其一，影像学手段在病理诊断前无法确定肿瘤的良恶性，会产生假阳性结果，再有影像学手段检测不到 < 3 mm-4 mm的结节，但 < 1 mm的癌变都可能与循环肿瘤细胞（circulating tumor cells, CTCs）的存在相关^[9]^；其二，重复进行影像学检查，多次暴露于电离辐射，会增加个体的患病风险，加重个体医疗负担，其经济效益也是值得考量的因素。虽然影像学可以在早期阶段检测到无症状和可切除的肺癌，但是迄今为止这种方法并不令人满意。

## 2 液体活检

组织病理活检是恶性肿瘤诊断的"金标准"^[10]^。随着组织学诊断的确立，现在通过荧光原位杂交（fluorescence *in situ* hybridization, FISH）或下一代测序（next-generation sequencing technology, NGS）来检测突变位点已成为一种趋势。然而，组织活检具有一定侵入性且操作起来相对复杂，只能反映肿瘤在某一时刻的状态^[11]^。由于肿瘤异质性的存在，这样可能会错过重要的肿瘤特征或突变信息，并且体积小的肿瘤可能还需要进行多次操作来获取足够的活检组织。再有获取的肿瘤组织的量可能仅足以用于病理评估，却不足以用于分子分析^[12]^。以上都是组织病理活检存在的不足。理想的检测手段需要能够对肿瘤的生物特性以及异质性进行准确地分析、检测样本易于收集、具有一定的成本效益等特点，从而有利于疾病的动态监测，以血液或其他体液样本为基础的非侵入性技术（液体活检）已经在这种情况下证明了其有效性^[13]^。可作为肺癌筛查或早期诊断的无创方法^[14, 15]^。

血基生物标志物的想法并不新鲜。几十年来，肿瘤蛋白质生物标志物如甲胎蛋白（α-fetoprotein, AFP）、癌胚抗原（carcino-embryonic antigen, CEA）、癌胚抗原19-9（carcinoembryonic antigen 19-9, CA19-9）等已广泛用于肺癌的筛查诊断、疗效评估、术后检测、预后判断等方面。这些标志物的实用性在临床实践中已经得到了充分的证实，但是由于其在非肿瘤条件下仍可升高，因此缺乏一定的特异性。于是越来越多的科研人员将目光投向了其他血基标志物的研究，它们广泛包括循环肿瘤细胞（circulating tumor cells, CTCs）、循环肿瘤DNA（circulating tumor DNA, ctDNA）、微小核糖核酸（microRNA, miRNA）、外泌体（exosomes）、肿瘤血小板（tumor educated platelets, TEPs）^[15-24]^。随着技术的成熟与发展，它们的检测手段变得越来越快速准确。

## 3 循环肿瘤细胞

### 3.1 概述

1869年，澳大利亚籍医生Ashworth^[25]^首次提出CTCs的概念——原发灶或转移灶脱落进入外周血的肿瘤细胞，而这些肿瘤细胞可能经历了上皮间质转化，具有更强的流动性和侵袭性，更易粘附于血管壁进而穿透，最终产生远处转移。由于CTCs的完整性，因此可提供关于肿瘤形态或蛋白表达的信息^[26]^。虽然对CTCs的研究已经持续了一个多世纪，但其在各种恶性肿瘤中的潜在用途尚未完全实现。主要原因是CTCs在外周血中十分稀少，对CTCs的捕获无异于大海捞针，有赖于检测技术的发展。这也一直是CTCs科研探索中的瓶颈所在。

### 3.2 检测方式

CTCs的检测可以分为利用物理特性（大小、密度、弹性）的直接分离方法，利用CTCs的细胞膜或者细胞内部的特殊物质（上皮细胞粘附分子、特异性抗体、酶类）进行的间接的分离检测方法^[19, 20, 27, 28]^，以及近期研发的新型检测技术，如磁性纳米网络技术（magnetic nanowire networks）^[29]^和我国拥有自主产权的oHSV1-hTERT-GFP法^[30]^。

癌细胞一般比正常细胞大。Rarecells和ScreenCell系统是基于尺寸差异的两种装置，具有特定孔径的过滤器来捕获CTCs^[31]^。利用恶性细胞在大小和形态上的差异^[32]^，微椭圆过滤器允许较小的可塑性细胞通过，而留下较大的不规则CTCs，对比下面介绍的CellSearch，该方法可以收集到上皮细胞粘附分子（epithelial cell adhesion molecule, EpCAM）阴性细胞，不足是难以捕获较小的CTCs。在诊断和筛查肺癌方面，膜过滤法（isolation by size epithelial tumor cells, ISET）无疑是最早并且最引人关注的方法^[21, 33, 34]^。在可切除非小细胞肺癌（non-small cell lung cancer, NSCLC）中，应用CellSearch可在19%-39%的患者中检出CTCs，ISET方法却能在36%-50%患者中检出CTCs^[31]^。基于微珠（micro-beads）的ISET方法可以增加CTCs的捕获纯度，1 mL/min的流速便从全血样品中分离出高达91%的靶细胞^[35]^。

CellSearch系统检测是一项结合富集分离与检测为一体、自动化程度较高的技术，是美国食品药品管理局（Food and Drug Administration, FDA）最早批准用于临床的检测方法。其利用针对各种上皮靶标的铁粒子和抗体，如EpCAM和细胞角蛋白（CK8、CK18和CK19）来鉴定CTCs。CellSearch虽然高度可靠，但在某些良性和炎性疾病中可能产生假阳性结果^[36]^。应用该方法在NSCLC中CTCs的检出率低于其他肿瘤^[36]^；由于存在上皮-间叶转换（epithelial mesenchymal transitions, EMT），基于EpCAM的富集技术可能会忽略这些与EMT相关的CTCs^[37]^，因此EpCAM阳性的CTCs在NSCLC中检出几率也较低^[38]^。目前该系统尚未被批准用于NSCLC诊断^[36, 39]^。可能还需要在CTCs捕获中加入多种标志物，以提高NSCLC患者中CTCs检测的敏感性。

磁性纳米网络技术（magnetic nanowire networks）^[29]^是由来自韩国的科研团队所研发的，建立的网络上表征了生物素化阳离子聚乙烯亚胺（biotinylated cationic polyethylenimine）和生物素化抗体鸡尾酒共轭磁性聚吡咯（biotinylated antibody cocktail-conjugated magnetic polypyrrole），能够以极为灵敏的方式提取循环游离DNA（circulating free DNA, cfDNA）和CTCs，提取量和提取纯度均十分可观。进而可以用于扩增癌症相关的罕见突变。

端粒酶在正常人体细胞中的活性被抑制，而在90%以上的肿瘤细胞中处于活化状态，因此可以利用检测端粒酶活性的方式来示踪CTCs。利用插入人端粒酶启动子和绿色荧光蛋白（green fluorescent protein, GFP）基因的单纯疱疹病毒（oHSV1-hTERT-GFP）作为示踪手段，对外周血中的CTCs进行捕获。该方法^[30]^是由我国医学科学院自主研发的检测手段，此法在检测CTCs的敏感性、可重复性和稳定性方面均值得肯定。

### 3.3 用于早期诊断的价值

有研究^[21]^表明，CTCs可以从胸部CT扫描时没有发现结节的肺癌高风险人群（慢性阻塞性肺病吸烟者）中分离出来。随后对该人群每年进行一次CT扫描，发现最初检测到CTCs的患者的肺部结节与肺腺癌相关。在该人群中，与初次存在CTCs相关的继发性肺癌的预测价值是100%^[21]^。可见胸部结节的患者中分离出的CTCs与已经诊断为肺癌的患者具有相同的恶性细胞形态学特征^[21, 34, 40, 41]^。但此类研究通常仅在病人数量较少的单中心环境下施行^[21]^；CTC的表征仅基于形态学，未在分子水平上进行进一步的分析。作为这项初步研究的后续行动，2015年12月启动了一项多中心研究，旨在纳入超过55岁的600名吸烟者，吸烟量超过每年30包并且为慢性阻塞性肺病患者。这些患者在3年内每年进行一次ISET检测和胸部CT扫描。对检测到的CTCs的相关研究也将同时进行。该项目（名为AIR项目，全称是circulating tumor cells as a potential screening tool for lung cancer）应于2019年1月完成。

### 3.4 优势与局限性

从外周血分离CTC，避免了侵入性和复杂的活检程序。肿瘤细胞系的培养需要的时间较长且均为同质，不能准确反映遗传多样性和不断变化的肿瘤微环境。与之相比，CTCs衍生的异种移植物可以更准确地反映癌症的生物学特征，为研究癌症的动态演变提供一个可视窗口，允许在分子水平上对肿瘤纵向演变进行监测，从而指导诊断、预后和治疗决策。

CTCs作为早期诊断的标志也存在一定的限制，合理有效的富集方法就是最为重要也急需攻克的难题。主要的挑战是如何获得足够数量的、最佳条件的、可用于进一步评估的CTCs。再有评估CTCs分子特征的技术仍然在不断发展，如何将其标准化，以应用到临床日常实践。

## 4 循环肿瘤DNA

### 4.1 概述

1940年Mandel和Metais首次提出了细胞外游离核酸的概念，ctDNA于1977年首次被发现，由于基因测序技术的快速发展，近几年得到了更广泛的关注。坏死或凋亡的细胞释放到血液中的DNA，被称为循环游离DNA（circulating free DNA, cfDNA），而在肿瘤患者的血液中，一部分cfDNA来自死亡的肿瘤细胞，这部分DNA就被定义为ctDNA^[42]^。ctDNA为单链或双链DNA^[43]^。当肿瘤负荷增加时，患者的ctDNA水平会升高^[44, 45]^。迄今为止，ctDNA是获得癌症患者血液中分子肿瘤相关改变的诊断、预后、以及预测信息的最佳材料^[46]^。Szpechcinski等^[47]^的研究显示，NSCLC患者血浆ctDNA水平不仅高于健康人群，而且也高于慢性呼吸道炎症疾病患者，更加体现了其在肺癌筛查和早期诊断中潜在的临床价值。

### 4.2 检测方式

研究^[48]^显示血清和血浆标本中的ctDNA含量不同。据报道血浆是ctDNA较好的来源^[49]^。血清中的cfDNA虽然比血浆多，但血浆中的突变载荷更高。因此可以对配对的血浆/血清样品同时分析，有助于提高分析灵敏度^[50-52]^。

应用荧光定量PCR（real-time polymerase chain reaction, RT-PCR）、数字PCR（digital PCR）等技术可以对ctDNA进行定量检测。基于小珠（Bead）、乳浊液（Emulsion）、扩增（Amplification）、磁性（Magnetic）这四个主要组分构建的BEAMing技术和通过深度测序进行癌症个性化分析的CAPP-seq（cancer personalized profiling by deep sequencing）技术已经改变了检测ctDNA的格局。这些技术通过使用已知的标签测序引物来扩增目标DNA。其中CAPP-seq可以鉴定100% Ⅱ期-Ⅳ期和50% Ⅰ期NSCLC患者的突变^[53]^，但必须已知目标基因，才能对其进行定量分析。

许多敏感的检测方法（如全基因组或外显子组测序）已经开发出专注于选择特定疾病中的特定基因。NGS可以在多个靶向基因组区域内同时进行测序，并且周转时间更短，样品需求也相对较少，因此被越来越多地用于ctDNA的分析当中。设计在Ion Torrent PGM平台（Thermofisher）中关于cfDNA常规诊断的有限NGS平台（命名为SiRe的定制基因组），可以靶向568个临床相关的突变基因，并且具有较高的诊断灵敏度和特异度，与其他数字或非数字检测平台相当^[51]^。

### 4.3 用于早期诊断的价值

ctDNA可以通过微创的方式获得，并且可以反映肿瘤组织中基因的突变。由于ctDNA的含量较少，有研究通过检测cfDNA中的特定突变来反映ctDNA的突变^[54]^。评估了51例小细胞肺癌（small cell lung cancer, SCLC）病例和123例非癌症对照血浆中提取的cfDNA中*TP53*突变的存在。在49%的SCLC患者的cfDNA中检测到*TP53*突变，11.4%的非癌症对照中检测到了突变，并且在35.7%早期SCLC患者cfDNA中检测到了*TP53*突变。提示ctDNA对癌症早期诊断的潜力是一个值得关注的领域。

### 4.4 优势与局限性

由于ctDNA可从血液样本中获取，与组织活检操作相比，可以降低患者的医疗风险和成本。ctDNA不仅可以阐明异质基因突变特征，还可以阐明疾病进展。ctDNA的突变和甲基化分析可能会提高癌症诊断的特异性。虽然ctDNA分析为早期肺癌诊断提供了一个可行的选择，但现存的技术仍无法克服敏感性分析的难关。再有如何将检测方法标准化是有待解决的问题。NGS本身的使用还涉及到一些问题，如成本、所需DNA的质量以及生物信息学支持的必要性等。

## 5 微小核糖核酸

### 5.1 概述

1993年Lee等首先在秀丽新小杆线虫中发现miRNA家族的第一个成员。miRNA是在真核生物中发现的一类内源性的具有调控功能的非编码RNA，其大小长约20个-25个核苷酸。miRNA通过介导转录后沉默在许多肿瘤类型中起关键作用^[55]^。加之已经证明，即使血浆在室温下长时间孵育，循环miRNA水平也可保持稳定^[56]^；经过多次冻融循环后也显示出了相应的抗性^[57]^。许多研究者一直致力于通过研究NSCLC患者、健康对照以及良性肿瘤患者中的循环或白细胞中的miRNA的差异表达来建立可靠的诊断工具。其中广泛研究的有let-7家族，在肺癌患者中常表现为低表达；在多项组合不同的miRNA进行肺癌筛查或早期诊断的研究中，常常都包含miRNA-21^[58, 59]^，其在肺癌患者中普遍高表达。

### 5.2 检测方式

miRNA的传统检测方法包括Northern blot、微阵列法（microarray）、RT-PCR，其中RT-PCR也是最常用、应用较为广泛的。传统方法存在操作繁琐、灵敏度低等缺点，因此许多灵敏度高、特异性好、简便快捷的检测手段应运而生。包括指数扩增反应（exponential amplification reaction, EXPAR）、滚环扩增技术（rolling circle amplification, RCA）、辅助酶目标核酸分子再循环策略（enzyme-assisted target recycling, EATR）等。还有新兴的数字PCR技术在检测微量miRNA上的表现也十分突出。根据最新的报道，Bo等^[60]^研发出了一项新型检测技术，基于靶向触发特异性双链环状核酸酶消化以及DNA-金纳米粒子的三重信号扩增测定miRNA的生物传感方案。记录电化学信号以呈现miRNA的初始水平。应用此方法已在肺癌患者中证实了miR-21的过表达，且与RT-PCR结果一致。该生物传感器不需要逆转录或任何PCR过程，满足了方便、快速、灵敏、特异的癌症早期诊断要求。该检测手段的潜力很大。

### 5.3 用于早期诊断的价值

Sozzi^[61]^和Montani等^[22]^鉴定了几种血浆miRNA，其在肺癌高风险人群中显示出了良好的预测价值。由34个miRNA组成的平台可以鉴别出一组无症状高危人群中的早期NSCLC患者，其准确性高达80%^[62]^。该平台有可能成为一种非侵入性的针对肺癌高危人群的筛查工具。

嗜中性粒细胞miRNA是用于检测早期肺癌的另一种生物标志物。中性粒细胞的功能可以通过调节特定基因的表达得以实现。Ma等^[63]^研究了中性粒细胞的miRNA水平及其与NSCLC的相关性。研究纳入了15例NSCLC Ⅰ期诊断患者和15例无肺癌诊断的吸烟者。NSCLC患者中存在5个miRNA表达差异。在验证人群中（共155例，包括肺癌人群和非肺癌人群），应用两种不同的miRNA检测NSCLC，敏感度为77.8%，特异度为78.1%；用来检测Ⅰ期和Ⅱ期NSCLC，敏感度分别为82.9%和68%，特异性均为77.6%。由此阐明嗜中性粒细胞miRNA谱可以用于发现早期肺癌。相关的*meta*分析^[64]^显示miRNAs在早期肺癌的诊断中Ⅰ期/Ⅱ期的敏感性、特异性、曲线下面积（area under curve, AUC）分别为0.81、0.82、0.88，Ⅰ期分别为0.80、0.81、0.88。一套特征性的miRNA可能成为筛查高危人群或诊断早期肺癌的有前景的生物标志物^[65]^。

### 5.4 优势与局限性

非侵入性和稳定性使得循环miRNA成为诊断癌症的潜在工具，其作为NSCLC生物标志物潜在作用的研究也是令人信服的。miRNA的局限性体现在用于定量检测的内参/外参基因的选择上存在不一致；不同来源的miRNA，如血浆、血清、全血、外泌体，在分离过程中存在质量和数量上的差异；部分研究的样本量较小，可能导致结果的不可靠。因此，对于miRNA的分离和定量，以及用于数据分析的方法仍然需要更多验证。

## 6 外泌体

### 6.1 概述

1983年，外泌体首次于绵羊网织红细胞中发现，1987年Johnstone将其命名为"exosome”。外泌体是一种细胞源性的囊泡，直径在30 nm-100 nm之间，它可以从血浆、唾液、尿液、乳汁、胸水、脑脊液、精液等多种体液中检测，其内含有蛋白质和miRNA等遗传物质，通过对肿瘤性外泌体的检测分析可以得到肿瘤细胞的相关信息^[66, 67]^。并且其在极端pH（pH=1-13）或冻融中稳定。因此，外泌体及其内容物可能成为诊断癌症的潜在生物标志物^[68]^。

### 6.2 检测方式

许多技术已经被开发用于外泌体的分离，如超速离心法、过滤离心、密度梯度离心法、免疫磁珠法、磷脂酰丝氨酸亲合法、色谱法等。分离后，Western blot、RT-PCR、核酸测序、ELISA、其他可商购的试剂盒可用于检测外泌体的蛋白质和RNA含量^[69]^。

### 6.3 用于早期诊断的价值

早期检测与肺癌相关的外泌体中的miRNA^[70]^。有研究^[71]^显示在肿瘤来源的外泌体的miRNA中，miR-181-5p、miR-30a-3p、miR-30e-3p和miR-361-5p体现了诊断腺癌的特异性，miR-10b-5p、miR-15b-5p体现了诊断SCC的特异性，通过NGS观察miR-320b，验证了其诊断的准确性。这些miRNA可能是开发高度敏感、非侵入性的针对早期NSCLC诊断的有效生物标志物的候选者。

### 6.4 优势与局限性

外泌体因具有囊泡包被，使得内部多种遗传物质可以稳定存在。循环外泌体miRNA与肿瘤衍生的miRNA之间的相似性使得循环外泌体miRNA可能用作肺癌的筛选试验。加之其内部的其他遗传物质，将会丰富肿瘤遗传学的相关研究。从初步获得的结果来看，其前景是十分可观的。然而获得外泌体的技术仍在发展当中，这也是限制外泌体研究的主要原因。

## 7 肿瘤血小板

### 7.1 概述

传统上以止血作用著称的血小板在肿瘤的生长发育过程中也起着显著的作用^[72, 73]^。TEP概念于2015年由Best提出^[73]^。在与肿瘤细胞对抗的过程中，肿瘤相关分子可以转移至血小板内，最终形成TEPs^[74, 75]^。当血小板表面受体得到激活^[76, 77]^，会发生特异性的前体mRNA剪接，从而产生可能用于癌症诊断的独特mRNA表达谱^[73]^。即未来TEPs可能被用作肺癌的血基生物标志物。

### 7.2 检测方式

应用标准差速离心法从全血中分离血小板，提取血小板RNA，扩增RNA随后进行测序。有研究^[78]^证明粒子群优化（particle-swarm optimization, PSO）增强算法能够从血小板RNA测序文库中有效地选择RNA生物标志物组。使得基于TEPs检测早期和晚期NSCLC的准确度得以提高（独立于个体年龄、吸烟习惯、全血存放时间、各种炎症反应）。

### 7.3 用于早期诊断的价值

一项比较癌症患者与健康受试者的血小板mRNA谱的研究^[73]^表明，与健康个体的血小板相比，TEPs中20种非蛋白质编码RNA的水平发生了改变，区分癌症患者和健康人的准确率达96%。患者的TEPs的RNA测序数据结合计算机的计算，判断样本的原发肿瘤部位的准确度为71%。在NSCLC患者中，TEPs的mRNA谱可以显示KRAS野生型肿瘤、*EGFR*突变型肿瘤、MET过度表达的*KRAS*突变型肿瘤患者的分化。

### 7.4 优势与局限性

较CTCs，肿瘤血小板更加丰富。但是目前分析的样本数量相对较少，算法过度拟合的风险较高^[73]^。获得TEP的技术仍在发展。

## 8 小结

目前大量的研究正在寻找一种理想的方法：其足够敏感、特异、可靠、可重复，可以用于早期诊断或预测肺癌的发展。液体活检似乎是最有希望的，可能成为胸部影像的补充或替代方法，用于肺癌的早期诊断和筛查。在效用和适用性方面正加速向临床应用发展。但液体活检仍存在一些局限性。一是分析前阶段存在的难度，对于血基生物标志物的研究来说，血液采样和分析阶段之间的延迟必须尽可能短；所有患者尽可能在相同条件下进行检测，才能展开更好的比较研究；采样必须用缓冲液调节血液，使研究中的目的生物标志物得到良好的保存。二是在肿瘤体积非常小或者在胸部影像学上不可见的患者中，其血液中的生物标志物的低量，这需要灵敏度高的检测技术。三是检测癌症特异性生物标志物，尤其是肺癌。虽然目前已经取得了令人鼓舞的结果，但是针对分析前的处理以及具体的分析步骤仍未得到标准化，这也是当前在临床实践中部署液体活检以用于癌症早期诊断的障碍。

一种有前景的方法是，即使影像学未检测到肿瘤，但是早期可以在血液中检测到相应的生物标志物。进行多中心研究、设立多学科团队（multidisciplinary team, MDT）、不同医学专业知识之间相互补充，在早期诊断和筛查的框架内进行液体活检，进而探索不同的循环生物标志物的研究范围和潜力。
